# Effects of Taping on Pain, Grip Strength and Wrist Extension Force in Patients with Tennis Elbow

**DOI:** 10.5812/traumamon.12450

**Published:** 2013-08-13

**Authors:** Alireza Shamsoddini, Mohammad Taghi Hollisaz

**Affiliations:** 1 Exercise Physiology Research Center, Baqiyatallah University of Medical Sciences, Tehran, IR Iran; 2 Department of Physical Medicine and Rehabilitation, Baqiyatallah University of Medical Sciences, Tehran, IR, Iran

**Keywords:** Hand Strength, Pain, Therapy, Elbow

## Abstract

**Background:**

Tennis elbow (TE) is a common musculotendinous degenerative disorder of the extensor origin at the lateral humeral epicondyle. Different modes of treatment are used for management of tennis elbow.

**Objectives:**

This study investigated the effect of the taping technique (TT) on pain, grip strength and wrist extension force in treatment of tennis elbow.

**Patients and Methods:**

Thirty patients (16 men /14 women with a mean age of 32.2 years) with tennis elbow of their dominant arm participated in this study. Outcome measures were assessment of pain at the lateral aspect of the elbow, grip strength and wrist extension force before and five to ten minutes after application of elbow tape on the affected and unaffected arms. A Visual Analog Scale was used to assess pain. A dynamometer and a hand-held dynamometer were used for evaluation of grip strength and wrist extension force, respectively.

**Results:**

Among the variables, significant differences were found in wrist extension forces between effected and unaffected arms (P = 0.02). Changes in grip strength showed statically significant improvements in the affected arm compared to the unaffected arm (P = 0.03). Also, in assessment of pain at the lateral epicondyle, the mean change between affected and unaffected arms was significant, with P = 0.001.

**Conclusions:**

The taping technique, as applied in this study demonstrates an impressive effect on wrist extension force and grip strength of patients with TE. Elbow taping also reduces pain at the lateral aspect of the elbow in these patients.

## 1. Background

Tennis elbow (TE) describes the condition Lateral Epicondylitis (LE), or inflammation of the outside portion of the elbow presenting with soreness and tenderness. Recent research has shown that TE is not as much inflammatory as it is degenerative (microscopic injury to the tendon). It commonly appears in a vast proportion of people who do not play tennis at all (1-3). LE is one of the most common causes of elbow and forearm pain encountered in clinical practice commonly associated with resistant wrist or finger extension and gripping activities (4-7).TE affects 1 to 3% of the adult population, occurs mainly as episodes in the dominant arm of patients aged 35 to 50 years, and is equally distributed between men and women (5, 8, 9). Recent systematic reviews of randomized clinical trials of a range of interventions, including friction massage, ultrasound, acupuncture, orthotic therapy, shock wave therapy, oral non-steroidal anti-inflammatory medication and surgery have indicated that the literature does not support many of the recommended physical treatments of TE (2, 10-14). McConnell has proposed the application of tape as a means of alleviating pain, improving muscle function, and restoring functional movement patterns. Clinically, in musculoskeletal conditions, by minimizing the aggravation of symptoms during the performance of therapeutic exercise, the use of a taping technique (TT) may facilitate compliance to exercise rehabilitation programs (15, 16). Elbow taping technique that was newly used for the treatment of tennis elbow may facilitate the compliance to exercise rehabilitation programs.

## 2. Objectives

The aim of the present study was to investigate the effect of taping technique on pain and grip strength and wrist extension of patients with TE immediately after application.

## 3. Patients and Methods

Thirty patients with TE (16 men and 14 women, mean age 32.2 years) of their dominant arm participated in this study. The patients were recruited through public advertisements and referrals from physical medicine and rehabilitation clinics. All participants underwent an initial assessment by a qualified musculoskeletal physiatrist and occupational therapist. The etiology of injury for all subjects was nonsport-related overuse. Subjects were selected through simple non-probability sampling. Inclusion criteria were: (a) pain experienced over the lateral epicondyle in at least one of the following tests: resisted static contraction of the wrist extensors or extensor carpi radialis brevis, or stretching of the extensor muscles of the forearm (8, 9); (b) involvement of one arm; (c) the patient had not been previously treated and (d) patient reporting unilateral LE for longer than 6 weeks. Exclusion criteria were: (a) dominant hand fracture during the previous year; (b) history of rheumatoid or neurologic disease; (c) limitation in the range of motion in the dominant hand and (d) radiculopathy. Ethical approval was granted for the study and all patients signed informed consent statements. 

**Figure 1.**
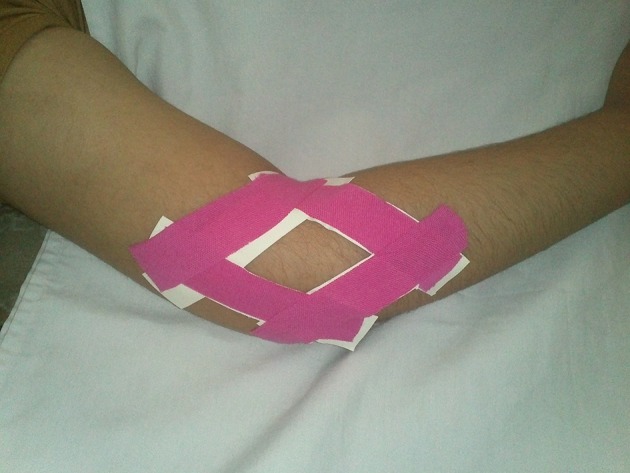
Taping Technique Used In the Current Study

Pain at the lateral epicondyle, grip strength and wrist extension force, with and without the use of elbow taping were examined. The unaffected arm served as a control. We used a dynamometer to measure grip strength in kilograms with the upper limb in a standardized position as recommended in a studies of elbow position and grip strength ( 17 , 18 ). The upper limb was positioned in a supine lying position; the palm of the hand was placed flat on the treatment table and adjacent to the subject’s side. Thus, there was an internal rotation of the shoulder, pronation of the forearm, 90° flexion of the elbow and slight shoulder abduction to allow for the dynamometer handles to fit between the hand and body ( 8 , 19 ). Wrist extension force was measured with a validated hand-held dynamometer (J Tech onsite, UK). We multiplied the output in kilograms by the lever arm distance from the third metacarpophalangeal (MP) joint to the radiolunate joint to derive a torque value ( 8 ). Pain was measured on a visual analogue scale (VAS), where 0 cm was the least pain imaginable and 10 cm was the worst pain imaginable ( 20 , 21 ). In this study, we used diamond taping. The diamond taping technique consisted of 4 pieces of approximately 80- to 100-mm long, 3.8 cm wide, non-elastic, adhesive tapes; these were applied on the skin distally to proximally in a diamond shape. In this study, we used a modified taping technique described in our previous study ( 22 ), where patients alone and without the help of the therapist, pasted tapes on their elbow. The strips overlapped at their ends and were secured with 4 additional tape strips (Figure 1). 

Duration of use of the tape in pretreatment (baseline) and (5 to 10 minute) post treatment were evaluated. Statistical analysis included independent sample t-test, used for the comparison of scores between affected and unaffected arms after taping. For assessment of taping technique in pre and post-intervention, mean scores were analyzed using a paired-sample t-test to determine whether there were any significant differences. Statistical analysis was performed with the SPSS software (version 17.0), with P-values less than 0.05 considered statistically significant.

## 4. Results

Based on the inclusion criteria, 16 men and 14 women with mean age of 32.2 years were enrolled in the study; 24 individuals were right-handed and 6 individuals were left-handed. Mean (± SD) duration of their tennis elbow condition was 6.4 ± 1.3 weeks. The data from this study demonstrated positive changes in grip strength with application of the taping technique on the affected arms when compared with the unaffected arm. This effect was observed within 5 to 10 minutes. For the affected arm, the maximum improvement in grip strength was on average 6.3 kilograms immediately after application; the maximum wrist extension force was on average 11.6 kilograms. The mean reduction of pain was 2.2; the data shows positive changes after application of the taping technique. For the unaffected arm, very little change in scores was demonstrated regarding grip strength (Table 1). 

**Table 1.** Change in Variables of Affected and Unaffected Arm

	Affected Arm		Unaffected Arm	
	Pre Application	Post Application	Pre Application	Post Application
**Pain at the lateral aspect of elbow**	4.2	2	0	0
**Grip strength, N**	31.2	37.5	39	40.1
**Wrist extension strength, N**	43.7	55.3	65.3	66.2

The results of the student t-test showed that there was a significant effect on grip strength between the effected and unaffected arm (P = 0.03). According to the result, the average difference wrist extension force between the affected and unaffected arm was significant (P = 0.02). Also, pain in the lateral aspect of the elbow was significantly different between the affected and the unaffected arm, immediately after application of taping (P = 0.001, Table 2). 

**Table 2.** Independent Sample t-test Analysis of Changes in Pain and Grip Strength and Wrist Extension Force

	Average Difference in Affected Arm	Average Difference in Unaffected Arm	P value
**Pain in the lateral aspect of elbow**	2.2	0	0.001
**Grip strength, N**	6.3	1.1	0.03
**Wrist extension Force, N**	11.6	0.9	0.02

## 5. Discussion

This study examined the immediate effect of taping on pain in the lateral aspect of the elbow and grip strength and wrist extension force in patients with TE. The data of our study demonstrated that the application of the taping improved grip strength immediately after application, in patients with tennis elbow. The significant differences in grip strength are comparable with the report by Vicenzino et al. in which the authors reported that using a diamond tape could significantly increase grip strength in patients with tennis elbow (23). Although, in our study, the mean increase in grip strength was not similar to Burton’s report (24), a major difference between studies was the test position used to measure grip strength. The current study and that by Wadsworth (25) and Wuori (26) tested grip strength in 90° of flexion, whereas the others had the elbow in extension (23). Participations demonstrated a statistically significant increase in wrist extension force while using tape only on the arm affected. This finding agrees with the results of the experiment by Wadsworth et al. (25) On the unaffected extremities of subjects wearing a forearm band. It also agrees with the experiment by Shamsoddini (22), who found that the tape can cause no increase in wrist extensor force of non-impaired subjects. We postulate that the significant increase in wrist extension force and grip strength of the affected arm occurred because taping disperses stresses generated by muscle contraction, thereby reducing painful inhibition and allowing the subject to contract more forcefully; although this has not been proven (25, 26). Data from our study also showed that an application of tape resulted in positive changes of pain scores compared with pre and post-application of tape. According to the results, data from our study was compatible with the study by Vicenzino in which the authors reported that the application of tape resulted in positive changes in pressure pain threshold scores compared to a placebo or no-tape controls (23). A possible model of the mechanism of action for taping in TE may relate to its neurophysiologic effects on the nervous system, particularly the nociceptive system. In this neurophysiologic model the tape may exert an effect on grip strength by primarily altering pain perception, either locally at the elbow by inhibiting nociceptors, facilitating large afferent fiber input into the spinal cord and/or possibly by stimulating endogenous processes of pain inhibition (4, 22, 23).

The data suggest that this method of treatment may be useful in the management of this condition when applied to the affected arm.
